# Burkitt lymphoma and lactic acidosis: A case report and review of the literature

**DOI:** 10.14814/phy2.14737

**Published:** 2021-02-21

**Authors:** Carole Looyens, Raphael Giraud, Ivo Neto Silva, Karim Bendjelid

**Affiliations:** ^1^ Intensive Care Division, Cardiovascular Unit Geneva University Hospitals Geneva Switzerland; ^2^ Faculty of Medicine University of Geneva Geneva Switzerland; ^3^ Geneva Hemodynamic Research Group Geneva Switzerland

**Keywords:** Burkitt lymphoma, lactate, pathophysiology

## Abstract

Type A lactic acidosis is a potentially life‐threatening complication in critically ill patients and is the hallmark of a shock state as a result of tissue hypoperfusion and dysoxia. Type B lactic acidosis results from mechanisms other than dysoxia and is a rare condition in patients with solid tumors or hematological malignancies. We present a case of a 60‐year‐old man with lactic acidosis who was found to have a Burkitt lymphoma related to a posttransplant lymphoproliferative disorder. Lactagenic cancers are characterized by increased aerobic glycolysis and excessive lactate formation, a phenomenon described by Warburg in 1923 that is correlated with cancer aggressiveness and poor survival. There is increased glucose utilization with the purpose of lactagenesis under fully oxygenated conditions, as lactate seems to be a potent signaling molecule for angiogenesis, immune escape, cell migration, metastasis and self‐sufficient metabolism, which are five essential steps of carcinogenesis. Type B lactic acidosis in association with malignancies carries an extremely poor prognosis. Currently, effective chemotherapy seems to be the only hope for survival.

## Graphical abstract

Lactagenic cancers are characterized by increased aerobic glycolysis and excessive lactate formation, a phenomenon described by Warburg in 1923 that is correlated with cancer aggressiveness and poor survival. Type B lactic acidosis in association with malignancies carries an extremely poor prognosis. Currently, effective chemotherapy seems to be the only hope for survival.

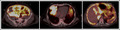

## 1 INTRODUCTION

A high arterial lactate level in critically ill patients has been associated with significant morbidity and mortality ever since the first description two centuries ago (Kompanje et al., 2007). Hyperlactatemia in the critically ill is the hallmark of shock states (Kraut & Madias, 2014; Levy, 2006; Nichol et al., 2010; Vincent & De Backer, 2013; Vincent et al., 2016), and the degree of increase in arterial lactate concentrations is directly related to the severity of the shock state (Haas et al., 2016; Nichol et al., 2011; Vincent et al., 2016). In this regard, the prognostic value of arterial lactate levels seems to be independent of the underlying critical illness (Jansen et al., 2010). Serial lactate measurements are widely used in intensive care medicine in the evaluation of the progression of a shock state and the response to intensive and urgent therapy (Levy et al., 2018; Vincent et al., 2016).

Although high lactate levels have been widely used as a marker of altered tissue perfusion in critically ill patients, this condition does not always simply reflect the development of anaerobic metabolism and cellular dysoxia better known as Type A lactic acidosis (Kraut & Madias, 2014; Vincent et al., 2016). While a lack of oxygen forbids the continuation of oxidative phosphorylation in the Krebs cycle, a normal oxygen supply does not impose a complete cessation of anaerobic metabolism. Type B lactic acidosis results from mechanisms other than dysoxia, including inborn errors of metabolism, drugs and toxins, systemic diseases (i.e., diabetes and sepsis), and less commonly malignancy. In this case report, we try to emphasize the pathophysiology leading to hyperlactatemia, and we will focus on the hyperlactatemia caused by lactagenic cancers and the Warburg effect.

## 2 CASE SUMMARY

A 60‐year‐old‐male patient presented at the emergency department with increasing abdominal girth, abdominal discomfort, severe asthenia, malaise, and profuse diaphoresis without fever. His medical history highlighted chronic renal failure (stage G3bA1) and a cardiac transplant 4 years ago due to terminal ischemic cardiomyopathy. His recent cardiac biopsy was free from any signs of rejection. He was taking immunosuppression with cyclosporine and mycophenolic acid. On admission, the clinical examination revealed a tense and distended abdomen with abdominal ascites. Initial vital signs were stable except for tachycardia at 110 beat per minute. The respiratory rate was 22 breath per minute, and the temperature was within the normal range. Physical exam was otherwise normal.

Laboratory analysis showed leukocytosis with a left shift, signs of hepatocellular injury without cholestasis, and chronic renal failure. His white blood count count was 14.8 G/L, C‐reactive protein was 45 mg/L (normal <10 mg/L), aspartate aminotransferase (AST) was 144 IU/L (normal, 14–50 IU/L), alanine aminotransferase (ALT) was 79 IU/L (normal, 12–50 IU/L), lactate dehydrogenase (LDH) was 1082 IU/L (normal, 87–210 IU/L), total bilirubin was 4 μmol/L (normal, 7–25 μmol/L), alkaline phosphatase was 82 IU/L (normal, 25–102 IU/L), gamma‐glutamyl transferase was 41 IU/L (normal, 9–40 IU/L), creatinine was 198 µmol/L (normal, 62–106 µmol/L), with an estimated glomerular filtration rate of 31 ml/min/1.73 m^2^. An ultrasound assessment confirmed the presence of moderate ascites in all four abdominal quadrants. Ascites liquid puncture showed leukocytosis, but the liquid culture remained sterile. Abdominal CT was then performed following a rapid clinical deterioration for abdominal sepsis, showing signs of diffuse peritonitis with a pelvic abscess located adjacent to the small intestine. The patient was therefore immediately started on broad‐spectrum antibiotics and antifungal treatment. In addition, a median laparotomy was performed to remove the pelvic abscess. The resected mass (suspect for any neoplasm) was sent for an extemporaneous analysis that elicited a high‐grade lymphoma. Later pathology testing confirmed a Burkitt lymphoma related to a posttransplant lymphoproliferative disorder. Following emergency department admission and immediate surgery, the patient was admitted to the intensive care unit for high anion gap lactic acidosis, with a pH of 7.29 and a lactate level of 7.4 mmol/L on the arterial blood gas analysis. However, the patient's vital signs remained stable, and he could be extubated on the same day. Signs of hypoxia or circulatory failure were absent, capillary refill time was within normal limits and there was no skin mottling. Moreover, lactate/pyruvate ratio value was 19.0. In contrast, he simultaneously developed profuse hypoglycemia necessitating continuous high‐dose intravenous glucose supplementation. Cardiac output measured with bedside echocardiography appeared to be within the normal ranges. A new postoperative abdominal CT angiography was performed to exclude abdominal ischemia. The lactic acidosis (mean pH 7.3) remained for several days with lactate levels fluctuating around approximately 7 mmol/L. A concomitant thiamine deficiency was excluded by intravenous supplementation. Lactic acidosis started to decrease once chemotherapy with cyclophosphamide and vincristine, initiated 5 days after surgical resection, began to have an effect.

**FIGURE 1:**
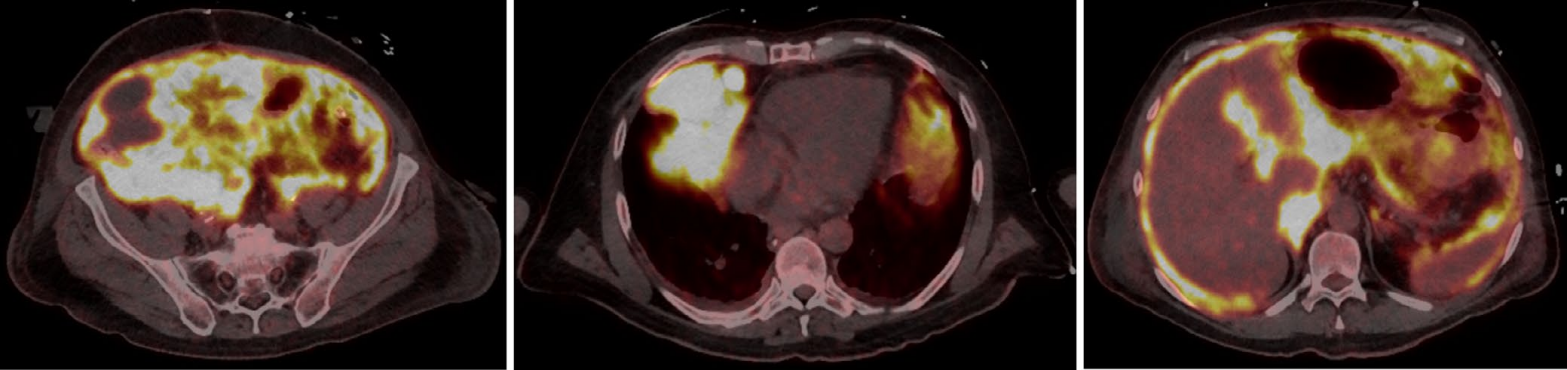
Left: Transversal image from patient's abdominal positron emission tomography–computed tomography (PET‐CT) post tumoral resection revealing diffuse peritoneal hypermetabolism with difficult differentiation between an infectious versus an oncological process. Middle: Transversal image from patients thoracic PET‐CT revealing supradiafragmatic invasion of the lymphoma with a mass and a nodule located in the right cardiophrenic recess with diffuse bilateral pleural nodular thickening. Right: Transversal image from patient's abdominal PET‐CT showing retroperitoneal adenopathies.

**FIGURE 2:**
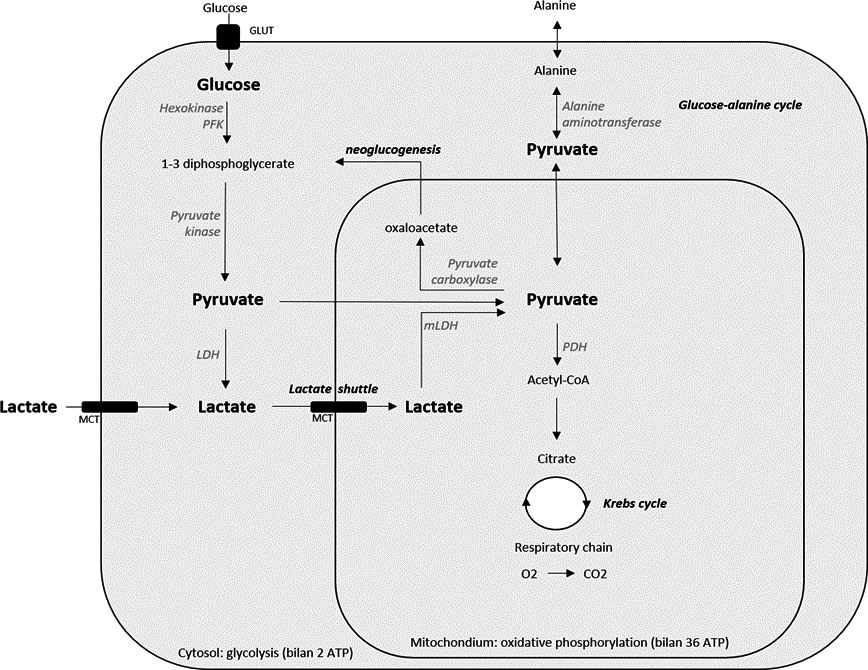
Lactate production and destination. Glycolysis takes place in the cellular cytosol. The preparatory phase consists of the generation of 1–3 diphosphoglycerate by hekoxinase and phosphofructokinase (PFK), the rate‐limiting enzyme. The pay off phase consists of the generation of pyruvate by pyruvate kinase. Pyruvate can be reduced to lactate by lactate dehydrogenase (LDH) or it can be converted to acetyl‐Co A by pyruvate dehydrogenase (PDH) and enters the mitochondrial Krebs cycle for further oxidative phosphorylation and energy production. Pyruvate can also undergo carboxylation into oxaloacetate, thereby initiating neoglucogenesis, or undergo a transamination by alanine aminotransferase, resulting in the formation of alanine. Lactate either locally formed after reduction of pyruvate or coming from a distant source, can be consumed in the mitochondria by reformation of pyruvate by mitochondrial lactate dehydrogenase (mLDH) after diffusion across membranes via monocarboxylate transporters (MCTs). This concept is better known as the cell‐to‐cell lactate shuttle.

**FIGURE 3:**
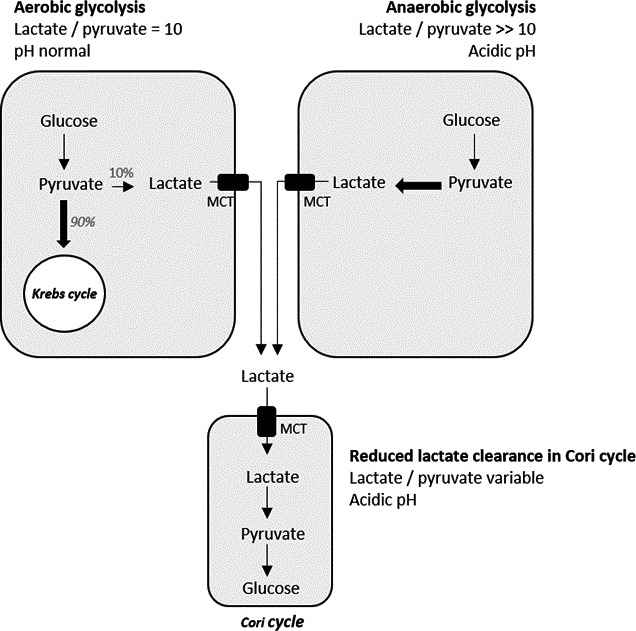
Lactate/pyruvate ratio. In aerobic glycolysis, the physiologic ratio of lactate/pyruvate is approximately 10 and is warranted by lactate dehydrogenase (LDH) catalyzing the reduction of pyruvate in lactate. When there is any perturbation of tissular oxygenation, cellular oxygenation is inhibited causing and acceleration of the reduction in pyruvate into lactate by LDH which results in a pathological lactate/pyruvate ratio above 10. The inhibition of the oxidative phosphorylation avoids any proton recycling leading to high anion gap lactic acidosis. Lactate is mostly converted into glucose through neoglucogenesis in the Cori cycle taking place in the liver and the kidneys. In case of insufficient hepatic perfusion or hepatic failure, lactate will be less cleared causing a variable lactate/pyruvate ratio depending on the source of production of lactate. Protons, normally recycled in the Cori cycle, will accumulate causing acidosis. MTC, monocarboxylate transporter.

The interpretation of an initial positron emission tomography–computed tomography (PET‐CT) (Figures 1, 2, 3) 6 days after surgery was not clear due to the difficult differentiation between an infectious versus an oncological process. In addition to diffuse hypermetabolism in the peritoneal cavity associated with retroperitoneal adenopathies, it revealed supradiaphragmatic invasion of the lymphoma with a mass located in the right cardiophrenic recess, left mammary adenopathies, and pleural and pericardial nodules. There were also two hypermetabolic, osseous spots, suspected of metastasis, one in the second cervical vertebrae and one in the left scapula.

He underwent intensive inpatient chemotherapy with one cycle of R‐CHP and six cycles of R‐CHOP (R = rituximab, C = cyclophosphamide, H = doxorubicin hydrochloride, O = vincristine sulfate, P = prednisone) and 10 prophylactic intrathecal injections of methotrexate. Chemotherapy was complicated with multiple episodes of febrile agranulocytosis, anemia and thrombocytopenia, which resolved after treatment with antibiotics in combination with filgrastim and multiple transfusions. The patient could be discharged with closed outpatient follow‐up after 5 months of admission. A PET‐CT was carried out 2 months after discharge and showed complete regression of all the lesions. A thoraco‐abdominal CT performed 1 year after the diagnosis seemed to be completely normal.

## 3 DISCUSSION

### 3.1 Lactate homeostasis

As with the blood levels of any substance, elevated lactate levels can be the result of increased production or reduced clearance, or both. Under physiological conditions, 1500 mmol of lactate or 20 mmol/kg of body weight is produced daily from various organs, including the muscle, intestine, red blood cells, brain, and skin (Kraut & Madias, 2014). Lactate is metabolized by the liver (60%), kidneys (30%), and other organs. The normal arterial blood lactate level is approximately 1 mmol/L (Kraut & Madias, 2014; Vincent et al., 2016).

### 3.2 Lactate production

Lactate formation is closely related to glycolysis. Glycolysis takes place in ten steps, five of which are in the preparatory phase and five in the payoff phase. Phosphofructokinase (PFK) is the rate‐limiting enzyme. Two net ATP molecules are generated by phosphorylation by high‐energy compounds. The final product of glycolysis is pyruvate and lactate, to which pyruvate can be reduced. LDH catalyzes the reduction of pyruvate into lactate at a well‐defined rate so that in normal homeostasis, the physiological ratio of lactate/pyruvate is approximately 10. In the presence of oxygen, pyruvate can be converted into acetyl‐coenzyme A by pyruvate dehydrogenase (PDH) and enters the mitochondrial Krebs cycle for further oxidative phosphorylation and energy production. This respiratory chain of reactions results in a net production of 36 ATP molecules per molecule of glucose. The metabolic yield of glycolysis when participating in the aerobic metabolic pathway (i.e., along with the Krebs cycle and oxidative phosphorylation) is superior to fermentation (without O_2_) into lactate (38 vs. 2 molecules of ATP). Anaerobic fermentation into lactate may be inefficient compared to oxidative phosphorylation, however, the rate of glucose metabolism into lactate is 10–100 times faster than the complete oxidation of glucose in the mitochondria (Liberti & Locasale, 2016). In fact, the amount of ATP synthesized over any given period of time is comparable when either form of glucose metabolism is utilized (Liberti & Locasale, 2016). In addition to the better‐known reduction to lactate and the oxidation to acetyl‐coenzyme A, for the completeness of this review, we need to mention that pyruvate can also undergo carboxylation into oxaloacetate, thereby initiating neoglucogenesis, or undergo a transamination by ALT, resulting in the formation of alanine (Ben‐Hamouda et al., 2013; Kraut & Madias, 2014).

Experiments by Pasteur (Platt, 1988), Meyerhof (1927), and A.V. Hill (Bassett, 2002) led to the widespread understanding of the glycolytic pathway and the notion that only a limitation of oxygen availability leads to fermentation and lactate accumulation. Out of this early work came the idea that lactate is just an anaerobic waste product that must be cleared from the muscles and blood, preferably by being converted to glucose in the liver via the Cori cycle. However, it has been demonstrated that lactate is a potent fuel and signaling molecule and is constantly being produced and circulated throughout the body, even and most often when there is adequate oxygen. Lactate is more than just a “hypoxic waste product.” Lactate as a result of dysoxia is often the exception rather than the rule, even in critically ill patients (Goodwin et al., 2014).

### 3.3 Lactate metabolism

Any time glycolysis is active, lactate is formed and equilibrates with local lactate gradients. Lactate equilibrates mainly by diffusing across membranes via monocarboxylate transporters (MCTs). In lactate‐producing tissues, this means exporting lactate into the circulation where both local and distant tissues can take it up and use it.

Lactate is metabolized by the liver (60%), kidneys (30%) and other organs. Lactate can be used for gluconeogenesis by reformation of pyruvate and glucose in the Cori cycle, which takes place in the liver and kidneys. In addition, lactate can readily replace glucose as a fuel for almost all cells of the body (any cell with mitochondria) by reformation and subsequent oxidation of pyruvate in the mitochondria. Lactate clearance occurs in the heart, liver, skeletal muscle and even brain. This observation that lactate is constantly being produced and consumed formed the basis of the cell‐to‐cell lactate shuttle, an energy exchange hypothesis originally introduced by Brooks in 1984 (Brooks, 1986). Lactate seems to be a key intermediate metabolite in whole body metabolism (Ben‐Hamouda et al., 2013; Goodwin et al., 2014; Kraut & Madias, 2014).

### 3.4 Hyperlactatemia (Table 1)

#### 3.4.1 Elevated production of lactate

As mentioned earlier, normal lactate production is approximately 1 mmol/min or 1500 mmol/24 h, and the normal lactate level is approximately 1 mmol/L (Ben‐Hamouda et al., 2013; Vincent et al., 2016). Elevated lactate production can be the consequence of either an elevated pyruvate concentration (as seen in accelerated glycolysis, in elevated protein catabolism or through inhibition of PDH) or cellular dysoxia. Dysoxic lactic acidosis is better known as type‐A lactic acidosis, and all other nondysoxic causes of lactic acidosis are classified as type B according to the classification of Woods and Cohen (Ben‐Hamouda et al., 2013). Indeed, if lactic acidosis occurs in the context of apparently adequate tissue oxygenation and normal hemodynamics (i.e. normal blood pressure, normal volemia, normal blood oxygen, and oxygen‐carrying capacity), it is traditionally termed Type B lactic acidosis. In the present setting, the physiologic ratio value of lactate/pyruvate is around 10 or higher.

**TABLE 1:** Classification of hyperlactatemia according to Woods and Cohen

Type A: Hyperlactatemia associated with cellular dysoxia due to insufficient oxygen supply	
Stagnant dysoxia	Low cardiac output
	Redistribution of cardiac output at the expense of certain tissues
	Vascular occlusion
Dysoxia caused by elevated demand of oxygen	Convulsions
	Intensive exercise
Dysoxia caused by low oxygen carrying capacity	Anemic dysoxia
	Carbon monoxide intoxication
Hypoxic dysoxia (low PaO_2_)	
Cytotoxic dysoxia due to inefficient mitochondrial consumption of oxygen	Sepsis
	Cyanide intoxication
Type B1: Hyperlactatemia due to an underlying disease	
Accelerated glycolysis	Hyperglycemia
	Sepsis
	Endogenous catecholamines
	Lactagenic cancer
Elevated protein catabolism	Burn victims
	Severe septic shock
Acquired inhibition of pyruvate dehydrogenase (PDH)	Sepsis
	Thiamine deficiency
	Lactagenic cancer
Reduced lactate clearance	Reduced liver blood flow
	Hepatic failure
Type B2: Hyperlactatemia due to drugs and toxins	
Exogenous catecholamines (adrenaline, dobutamine, terbutaline)	Cocaine, metamphetamines
Propofol	Salicylates
Metformine	Antiretroviral drugs
Linezolid	Toxic alcohols (ethanol, methanol, propylene glycol, ethylene glycol)
Paracetamol	
Type B3: Hyperlactatemia due to inborn errors of metabolism (enzymatic deficiencies)	
Congenital PDH deficiency	
Glucose‐6‐phosphatase deficiency (von Gierke disease)	
Pyruvate carboxylase deficiency	
Methylmalonic aciduria	
Mitochondrial encephalomyopathies	

##### Accelerated glycolysis

Any cause of an accelerated level of glycolysis causes an expected elevation of pyruvate and thus of lactate, as the physiologic ratio of lactate/pyruvate of approximately 10 or higher is always warranted by LDH. Accelerated glycolysis is the case in hyperglycemia, sepsis, or other situations with elevated endogenous or exogenous catecholamines (Ben‐Hamouda et al., 2013; Kraut & Madias, 2014). Finally, accelerated aerobic glycolysis may partially explain the hyperlactatemia caused by tumoral cells of lactagenic cancers, known as the Warburg effect (San‐Millán & Brooks, 2017), the cause of lactic acidosis in the present case.

##### Elevated protein catabolism

Elevated protein catabolism can also cause a rise in pyruvate levels. As mentioned earlier, pyruvate can be transaminated into alanine, and the reverse can be witnessed through the activity of the ALT enzyme, forming pyruvate in the liver. This mechanism contributes to hepatic neoglucogenesis. Accelerated protein catabolism is seen as muscle wasting, present in many critically ill patients, most severely in burn victims and in patients with severe septic shock. The elevated alanine supply in the liver contributes to the elevated pyruvate concentration and, ultimately, to hyperlactatemia, as the physiologic lactate/pyruvate ratio is always warranted by LDH (Ben‐Hamouda et al., 2013).

##### Inhibition of PDH

As discussed earlier, pyruvate is oxidized into acetyl‐coenzyme‐A by PDH. A congenital or an acquired reduction in the enzymatic activity of PDH can cause an accumulation of pyruvate and therefore of lactate. An acquired reduction in PDH can be caused by certain endotoxins and inflammatory cytokines in sepsis. This explains why hemodynamically stable patients with sepsis and normal liver function may have lactic acidosis (Kraut & Madias, 2014). It can also be caused by nucleoside reverse transcriptase inhibitors used to treat patients with HIV or by a thiamine deficiency, as in patients receiving parenteral nutrition or with severe Beri‐beri. Thiamine is an important cofactor in the PDH complex. Without thiamine, this enzyme cannot convert pyruvate into acetyl coenzyme A, and instead, conversion into lactate takes place (Friedenberg et al., 2007). Finally, certain oncogenes express PDH kinase, which inactivates PDH and inhibits the Krebs cycle (San‐Millán & Brooks, 2017; Swenson, 2016) and partially explains the development of hyperlactatemia caused by tumoral cells.

##### Cellular dysoxia

Perturbations of tissular oxygenation, termed “cellular dysoxia”, are caused by insufficient oxygen supply. This dysoxia can be either generalized due to a low cardiac output, carbon monoxide intoxication, profound arterial hemoglobin desaturation, and reduced oxygen content or either localized in the context of redistribution of the cardiac output at the expense of certain tissues or due to a vascular occlusion (Swenson, 2016). It can also be caused by mitochondrial enzyme defects and by inhibitors of aerobic metabolism, such as cyanide. Every drop in cellular oxygenation causes an acceleration of the reduction in pyruvate to lactate by LDH, which results in a pathological augmentation of the lactate/pyruvate ratio above 10 (Ben‐Hamouda et al., 2013). Even if the pyruvate dosage is expensive, its use and the finding of a pathological L/P level confirm cellular dysoxia and excludes other causes.

#### 3.4.2 Reduced lactate clearance

Lactate is partly transported to the liver and the kidneys and converted to glucose through gluconeogenesis (the Cori cycle). The hepatic clearance of lactate thus depends on hepatic extraction and hepatic functioning. Hepatic extraction is determined by the liver blood flow, which needs no less than one‐fourth of its normal flow. The capture of lactate by hepatocytes depends on its transportation by a family of MCTs with different isoforms (MCT1‐4). Thus, neoglucogenesis will depend on hepatic functioning. Its activity is decreased in hepatic failure and inhibited in shock states and severe acidosis (Ben‐Hamouda et al., 2013; Kraut & Madias, 2014).

### 3.5 Distinction between hyperlactatemia and lactic acidosis

Hyperlactatemia and lactic acidosis are frequently mixed, which may cause confusion. However, the two are governed by a different concept. Glycolysis causes lactate formation without lactic acidosis when there is no net H^+^ production. The H^+^ protons may arise following ATP hydrolyzation and are produced through glycolysis. However, those protons are recycled by lactate consumption either through the Krebs cycle or through the hepatic Cori cycle, hence maintaining the internal acid‐base balance (Ben‐Hamouda et al., 2013; Kraut & Madias, 2014).

In this regard, acquired or congenital inhibition of PDH, inhibition of oxidative phosphorylation due to cellular dysoxia, medication or intoxication and hepatic insufficiency are all causes of reduced proton recycling in either the Krebs or the Cori cycle, therefore causing high anion gap lactic acidosis. Other causes of hyperlactatemia, such as elevated glycolysis in hyperglycemia, arising from beta‐adrenergic stimulation or muscular catabolism, will only cause an elevated production of lactate without proton recycling impairment and without ensuing concomitant lactic acidosis (Kraut & Madias, 2014).

### 3.6 Lactagenic cancers

#### 3.6.1 Warburg effect

In glycolytic tumors, lactate levels of cancer cells are markedly elevated up to 40‐fold and are highly associated with cancer aggressiveness and poor survival (Brizel et al., 2001; San‐Millán & Brooks, 2017). In fast‐growing malignancies, the rate of tumor metabolism may be great enough to exceed normal muscle and liver lactate clearance and cause systemic type B lactic acidosis (Sillos et al., 2001; Swenson, 2016). The majority of lactic acidosis in malignancies is reported in cases of hematologic malignancies. Only a few cases have been reported for solid tumors presenting with lactic acidosis (Nair & Shah, 2017).

In 1923, Warburg observed in his Nobel Prize winning studies that cancer cells were characterized by accelerated glycolysis and excessive lactate formation even under fully oxygenated conditions and that tumor cells live and grow in a more acidic milieu as a result of increased lactic acid production not generally tolerated by normal cells (San‐Millán & Brooks, 2017; Swenson, 2016). His discovery was named the “Warburg effect” by Racker in 1972 (San‐Millán & Brooks, 2017). The Warburg effect is a hallmark of cancer, and its significance is still apparent in the common cancer diagnostic test using fluorodeoxyglucose positron emission tomography, which has a high diagnostic accuracy (Potter et al., 2016).

From a contemporary perspective of cell metabolic efficiency, it seems difficult to understand why, despite fully aerobic conditions, cancer cells choose an inefficient pathway producing two ATP molecules per molecule of glucose instead of 38 via coupled mitochondrial respiration. However, as mentioned earlier, the amount of ATP synthesized over any given period of time is comparable in both ways of glucose metabolism because fermentation into lactate is 10–100 times faster than the completion of oxidative phosphorylation (Liberti & Locasale, 2016). Moreover, the stress of lactate production is simply passed on to the host. Then, the cancer cells proliferate, and the tumor grows and metastasizes because of host exploitation, ending in final host expiration (San‐Millán & Brooks, 2017).

#### 3.6.2 Origin of tumoral lactagenesis

Lactagenesis is a highly orchestrated effort from oncogenes and tumor suppressor mutations for continuous and unstoppable glucose utilization to produce lactate involving five major steps: (i) an increase in glucose uptake; (ii) upregulation of PFK, the rate‐limiting enzyme of glycolysis; (iii) a decrease in mitochondrial respiration by upregulation of PDH kinase (PDK) that inhibits pyruvate uptake in mitochondria; (iv) increased lactate production by LDH upregulation; and (v) upregulation of MCT1 and MCT4 expression, the transmembrane transporters of lactate, for higher lactate plus H^+^ efflux and further lactate shuttling, thus mediating tumor growth and proliferation (Brahimi‐Horn et al., 2011; San‐Millán & Brooks, 2017; Swenson, 2016).

In our case, the Warburg effect was caused by a Burkitt lymphoma, a B‐cell derived malignancy. The main characteristic of a Burkitt lymphoma is an increased production of the MYC oncoprotein caused by chromosomal rearrangements. This translocation results in inappropriately high expression levels of MYC, which gives cells proliferative capacity. Moreover, MYC also activates the transcription of genes encoding glucose transporter, hexokinase, MCT, PDK, PK (pyruvate kinase), and LDH, resulting in the accelerated aerobic glycolysis or the Warburg effect. The MYC protein is the master regulator of the Warburg effect in BL cells (Mushtaq et al., 2015).

#### 3.6.3 Aim of tumoral lactagenesis

The role of the Warburg effect in the pathogenesis of cancer has not yet been completely established. Lactate is the end product of the Warburg effect, but lactate production or lactagenesis is probably the purpose of the Warburg effect as well.

In addition to being a potent oxidative fuel, lactate is also a potent signaling molecule necessary for all the major steps in carcinogenesis, as follows: (i) angiogenesis, (ii) cell migration, (iii) metastasis, (iv) immune escape, and (v) self‐sufficiency of cancer cells (San‐Millán & Brooks, 2017).

Lactate released from tumor cells by MCT4 plays a role in stimulating *angiogenesis* (i) by increasing the expression of vascular endothelial growth factor protein in endothelial cells (Goodwin et al., 2014; San‐Millán & Brooks, 2017). *Cell migration* (ii) is another essential step in carcinogenesis and metastasis in which lactate seems to be a key element to increase cell migration. Marked extracellular acidosis appears to promote the migration and *metastasis* (iii) of cancer cells by disrupting normal cell‐matrix interactions that act to maintain stable growth patterns (Swenson, 2016). Lactate levels are highly associated with a high incidence of distant metastasis. Acidosis reduces host defense against malignant cells and contributes to *immune escape* (iv) in many different ways: first, by inhibiting the release of the cytokines tumor necrosis factor and interleukin‐6; and second, by inhibiting the activation of T‐cells with a decrease in the cytotoxic activity of T‐cells and inhibition of natural killer cell functioning. Lactate also plays a central role in the *self*‐*sufficiency and sustainability* (v) of cancer cells. Cancer cells at the hypoxic core might use glucose and produce lactate, whereas cells on the periphery, close to a robust vascular supply, might take up this lactate and oxidize it as a fuel. The self‐sufficiency depending upon high glycolytic flux also allows cancer cells to produce lactate for carcinogenesis by angiogenesis, immune escape, cell migration, and metastasis. A glucose to lactate shunt occurs in which the host bears the burden of providing a limitless glucose supply as well as a sink for disposal of lactate and hydrogen ions. This may even explain why the actual cause of cachexia and death due to cancer appears to be multifactorial, with organ failure rather than the tumor itself (San‐Millán & Brooks, 2017).

#### 3.6.4 Targeting lactate production and shuttling: Future direction in cancer treatment

Given the profound changes in acid‐base balance in tumors and the role of pH in tumor survival and growth, altering the acid‐base milieu has presented itself as an interesting approach for treating cancer (Swenson, 2016), but the development of these new adjuvant therapies goes way beyond the scope of this article. Nevertheless, after explaining all the pathophysiology, it seems important to shed light on some future possible treatments: on the one hand, medication increasing PDH activity, such as dichloroacetate, seems to halt carcinogenesis by lowering cytosolic lactate production; on the other hand, MCT1 and MCT4 inhibitors seem to have enormous potential in cancer treatment by inhibiting lactate shuttling, even if there is still a lack of specificity (San‐Millán & Brooks, 2017). Furthermore, simpler approaches such as aerobic exercise seem to have beneficial effects by augmenting mitochondrial size and function and thus lactate clearance capacity. Further research is necessary to identify possible targets and create tumor‐specific treatments.

#### 3.6.5 Treatment options in patients with type‐B LA due to malignancies

Lactic acidosis in association with malignancies carries an extremely poor prognosis with a mortality rate over 90% (Nair & Shah, 2017; Sillos et al., 2001). Moreover, the high mortality associated with lactic acidosis has prompted some oncologists to consider this an oncological emergency (Nair & Shah, 2017). The best treatment for patients with hematologic malignancies who develop type B lactic acidosis is not yet clear.

##### Chemotherapy

Initiating aggressive chemotherapy has been effective in correcting acute acidosis (Friedenberg et al., 2007). It is actually the only treatment modality that consistently leads to remission. Resolution of lactic acidosis was reported to occur as early as 15 h and up to 3 days after starting chemotherapy. This treatment would not be effective in patients whose tumors are unresponsive to chemotherapy. Lactic acidosis improves with chemotherapy, and resolution of the lactic acidosis could be a surrogate marker of inducing remission (Chan et al., 2009).

##### Intravenous bicarbonate

The use of IV bicarbonate as a treatment for profound acidosis has never shown a meaningful clinical benefit, even in the worst cases (Swenson, 2016). As severe acidosis can cause respiratory fatigue and hemodynamic instability, intravenous bicarbonate is often used to attenuate systemic acidosis and increase the responsiveness to catecholamines. However, it would appear that the benefits of sodium bicarbonate are outweighed by its disadvantages, such as hypernatremia and hyperosmolality (Swenson, 2016). Acidemia leads to unloading oxygen from hemoglobin by shifting the hemoglobin‐oxygen dissociation curve to the right, and reducing acidosis will hinder oxygen release. Studies have shown that intracellular acidosis tends to slow lactate production (Madias, 1986; Sillos et al., 2001). Alkalinization has been shown to potentiate lactate production in patients with malignancy‐induced chronic lactic acidosis (Fields et al., 1981; Fraley et al., 1980). The effect of IV bicarbonate on mortality or lactate concentration in the setting of malignancy‐induced type B lactic acidosis has not been studied directly, as the incidence is very low. In a case report by Fraley et al. (1980), administration of bicarbonate improved pH but not the serum bicarbonate level. Intravenous bicarbonate corrected the extracellular pH but did not affect the significant intracellular acid production due to high tumor cell turnover. The use of sodium bicarbonate may not be recommended in these patient groups.

##### Renal replacement therapy

Renal replacement therapy, continuous or intermittent, in patients with renal dysfunction may be useful in addition to chemotherapy to correct metabolic acidosis. Here, once again, since the prognosis of type B lactic acidosis related to malignancies is grim, the only chance for remission is starting cytoreductive chemotherapy. Intravenous bicarbonate and hemodialysis will no longer act as a bridge to stabilize the patient enough so that the underlying cause can be treated (Chan et al., 2009).

##### Intravenous insulin

Lactic acidosis has also been treated with *intravenous administration of insulin*, which increases the conversion of pyruvate to acetyl‐coenzyme A and consequently facilitates oxidation of lactate to pyruvate (Sillos et al., 2001). Administration of glucose can actually induce lactic acidosis by increasing the availability of glucose and thus increasing the production of lactate by the tumor. Returning to the case presented in this manuscript, the patient was severely hypoglycemic, which is the reason why we substituted for intravenous glucose to maintain the patient's euglycemia. However, in a provocative hypothesis in 2009, Nijsten & van Dam (2009) presented a hypothetical treatment whereby glucose might be systemically lowered. If tumors are glucose consumers and lactate producers and all other tissues in the body can actively take up and use lactate as a fuel, why not systemically induce hypoglycemia to starve tumor cells? In this configuration, lactate would provide salvage fuel for the other tissues (Goodwin et al., 2014). Work to investigate this concept should be pursued.

## 4 CONCLUSION

Lactic acidosis is a commonly encountered problem in intensive care units and is most commonly associated with dysoxia, better known as type A lactic acidosis. Type B is more uncommon and can be life‐threatening and sometimes even a lethal complication in patients with malignancies. Due to its rareness, it is likely to be under recognized and therefore underdiagnosed. If oncological patients develop high anion gap lactic acidosis without hemodynamic compromise associated with acute respiratory distress without a pulmonary source, the possibility for tumor‐induced type B lactic acidosis through the “Warburg effect” should be considered. Awareness about this condition is important in the clinical practice of intensive care physicians since it will allow a timely diagnosis and the implementation of subsequent therapy. Currently, effective chemotherapy seems to be the only hope for survival.

## CONFLICT OF INTERESTS

The authors declare that they have no competing interests.

## AUTHORS' CONTRIBUTIONS

Carole Looyens, and Karim Bendjelid designed the present review. Carole Looyens, and Karim Bendjelid analyzed data and references. Carole Looyens, Raphael Giraud, Ivo Neto Silva, and Karim Bendjelid wrote the manuscript. All authors read and approved the final manuscript.

## Data Availability

The data that support these findings are available upon reasonable request from the corresponding author.
